# Supplementary material for: Sex chromosome identification and genome curation from a single individual with SCINKD
Source: bioRxiv. 2025 Jul 10:2025.07.07.660342. Preprint. [Version 1] doi: 10.1101/2025.07.07.660342 (PMC12265721; doi:10.1101/2025.07.07.660342)

bioRxiv preprint doi: https://doi.org/10.1101/2025.07.07.660342; this version posted July 10, 2025. The copyright holder for this preprint (which was not certified by peer review) is the author/funder, who has granted bioRxiv a license to display the preprint in perpetuity. It is made available under aCC-BY 4.0 International license.

Supplemental materials

Supplemental Table 1: Sample list and accession numbers.

Group	Species	Genotype	Type	Cov.	Database Accessions
Squamata; Gekkota; Sphaerodactylidae	*Sphaerodactylus notatus*	XY	PacBio HiFi	20x	SAMN48008689
Squamata; Gekkota; Sphaerodactylidae	*Sphaerodactylus notatus*	XY	HiC	N/A.	SAMN48008690
Squamata; Gekkota; Diplodactylidae	*Correlophus ciliatus*	ZW	PacBio HiFi	30x	SRR28508818-22
Squamata; Gekkota; Diplodactylidae	*Correlophus ciliatus*	ZW	HiC	N/A.	SRR29774324
Squamata; Gekkota; Eublepharidae	*Eublepharis macularius*	N/A.	PacBio HiFi	30x	SRR21708437
Squamata; Gekkota; Eublepharidae	*Eublepharis macularius*	N/A.	HiC	N/A.	SRR21708436
Squamata; Gekkota; Gekkonidae	*Lepidodactylus listeri*	XY	PacBio HiFi	30x	DA149208, DA149210, DA162797
Squamata; Gekkota; Gekkonidae	*Lepidodactylus listeri*	XY	HiC	N/A.	HG5YLDMXY
Squamata; Scincomorpha; Scincidae	*Cryptoblepharus egeriae*	XY	PacBio HiFi	40x	DA149190, DA149222
Squamata; Scincomorpha; Cordylidae	*Hemicordylus capensis*	XY	PacBio HiFi	43x	SRR22311010-12, SRR22311021
Squamata; Scincomorpha; Cordylidae	*Hemicordylus capensis*	XY	HiC	N/A.	SRR22311007-8
Mammalia	*Pan troglodytes*	XY	Genome	60x	GCF_028858775.2, GCA_028858805.2

bioRxiv preprint doi: https://doi.org/10.1101/2025.07.07.660342; this version posted July 10, 2025. The copyright holder for this preprint (which was not certified by peer review) is the author/funder, who has granted bioRxiv a license to display the preprint in perpetuity. It is made available under aCC-BY 4.0 International license.

Mammalia; Carnivora	*Vulpes vulpes*	ZZ	Genome	28x	GCA_964106825.2, GCA_964106925.2
Aves; Columbidae	*Caloenas nicobarica*	ZZ	Genome	44x	GCA_036010745.1, GCA_036013445.1
Aves; Columbidae	*Guaruba guarouba*	ZW	Genome	52x	GCA_045345405.1, GCA_045519325.1
Squamata; Lacertidae	*Podarcis gaigeae*	ZW	Genome	44x	GCA_964106915.2, GCA_964106785.1
Squamata; Lacertidae	*Podarcis cretensis*	ZZ	Genome	N/A.	GCA_951804945.1
Squamata; Lacertidae	*Podarcis cretensis*	ZZ	PacBio HiFi	27x	ERR11413981, ERR11413982
Chondrichthyes; Narcinidae	*Narcine bancroftii*	XY	Genome	50x	GCF_036971445.1, GCA_036971175.1

bioRxiv preprint doi: https://doi.org/10.1101/2025.07.07.660342; this version posted July 10, 2025. The copyright holder for this preprint (which was not certified by peer review) is the author/funder, who has granted bioRxiv a license to display the preprint in perpetuity. It is made available under aCC-BY 4.0 International license.

Supplemental Figure 1: Preliminary data suggesting that the number of biallelic SNPs across individuals roughly correlates with chromosome size in humans using whole genome re-sequencing (WGS) in GTEx data. In addition, the X chromosome contains fewer SNPs than expected based on its length, which is the approximate length of chr7 in GRCh38.

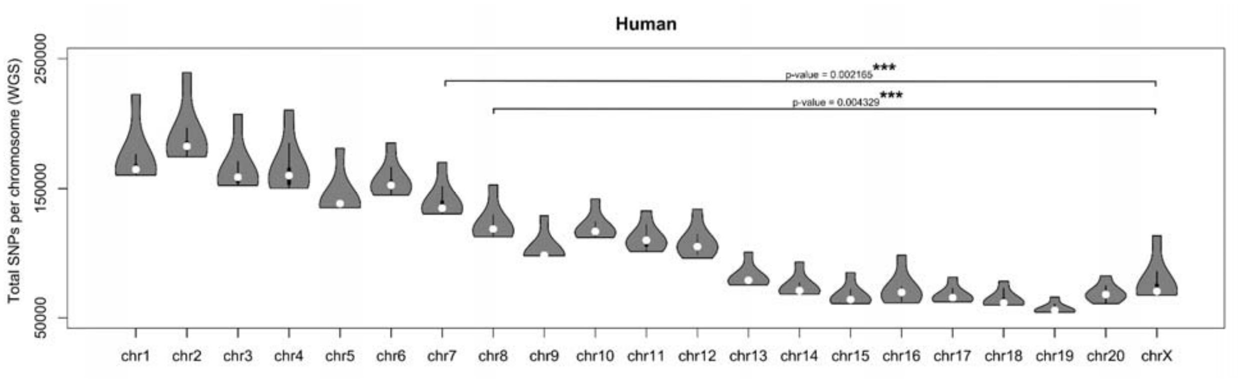

bioRxiv preprint doi: https://doi.org/10.1101/2025.07.07.660342; this version posted July 10, 2025. The copyright holder for this preprint (which was not certified by peer review) is the author/funder, who has granted bioRxiv a license to display the preprint in perpetuity. It is made available under aCC-BY 4.0 International license.

Supplemental Figure 2: Confirmation of a strong correlation (R^2 == 0.901) between hap-mers and chromosome length in a temperature-dependent species, the leopard gecko (*Eublepharis macularius*). Subtle variations between haplotypes are thought to be driven by poor phasing performance driven by low heterozygosity and/or sub-optimal coverage (30x) of PacBio HiFi data (Pinto et al. 2023).

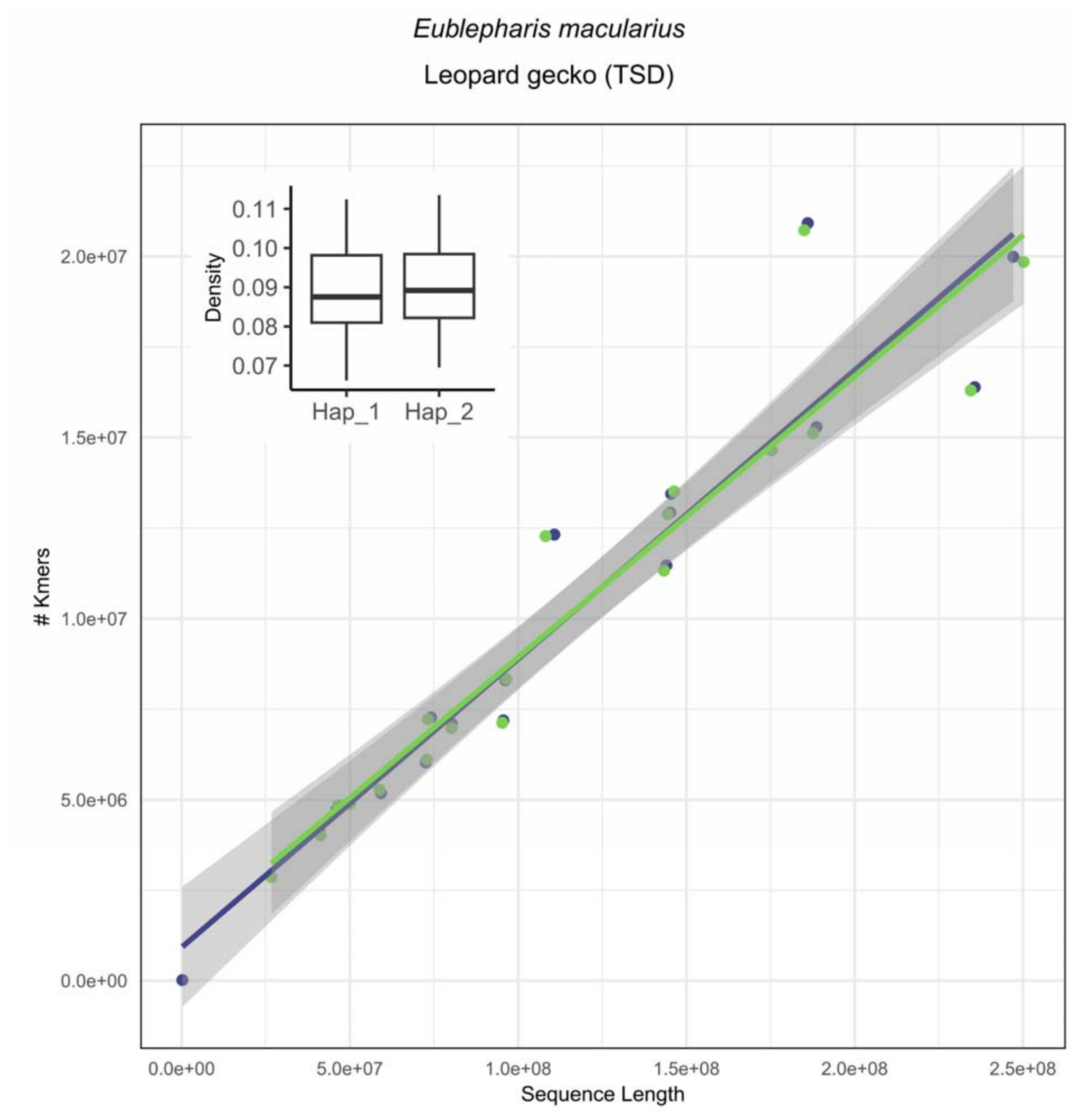

bioRxiv preprint doi: https://doi.org/10.1101/2025.07.07.660342; this version posted July 10, 2025. The copyright holder for this preprint (which was not certified by peer review) is the author/funder, who has granted bioRxiv a license to display the preprint in perpetuity. It is made available under aCC-BY 4.0 International license.

Supplemental Figure 3: Mirror plots for heteromorphic systems detailed in Figure 2.

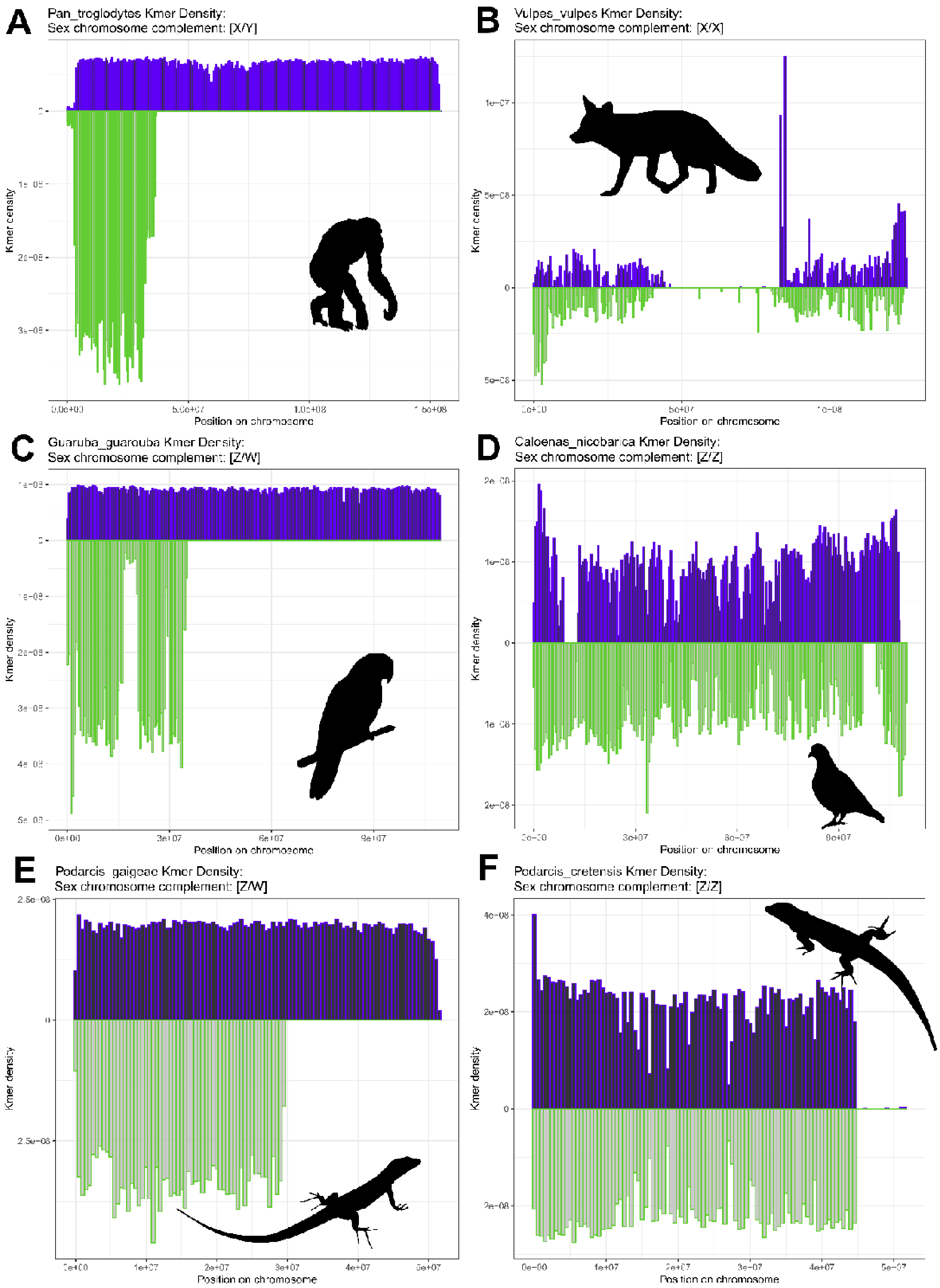

bioRxiv preprint doi: https://doi.org/10.1101/2025.07.07.660342; this version posted July 10, 2025. The copyright holder for this preprint (which was not certified by peer review) is the author/funder, who has granted bioRxiv a license to display the preprint in perpetuity. It is made available under aCC-BY 4.0 International license.

bioRxiv preprint doi: https://doi.org/10.1101/2025.07.07.660342; this version posted July 10, 2025. The copyright holder for this preprint (which was not certified by peer review) is the author/funder, who has granted bioRxiv a license to display the preprint in perpetuity. It is made available under aCC-BY 4.0 International license.

Supplemental Figure 4: Single sex-only mirror plots (part 1).

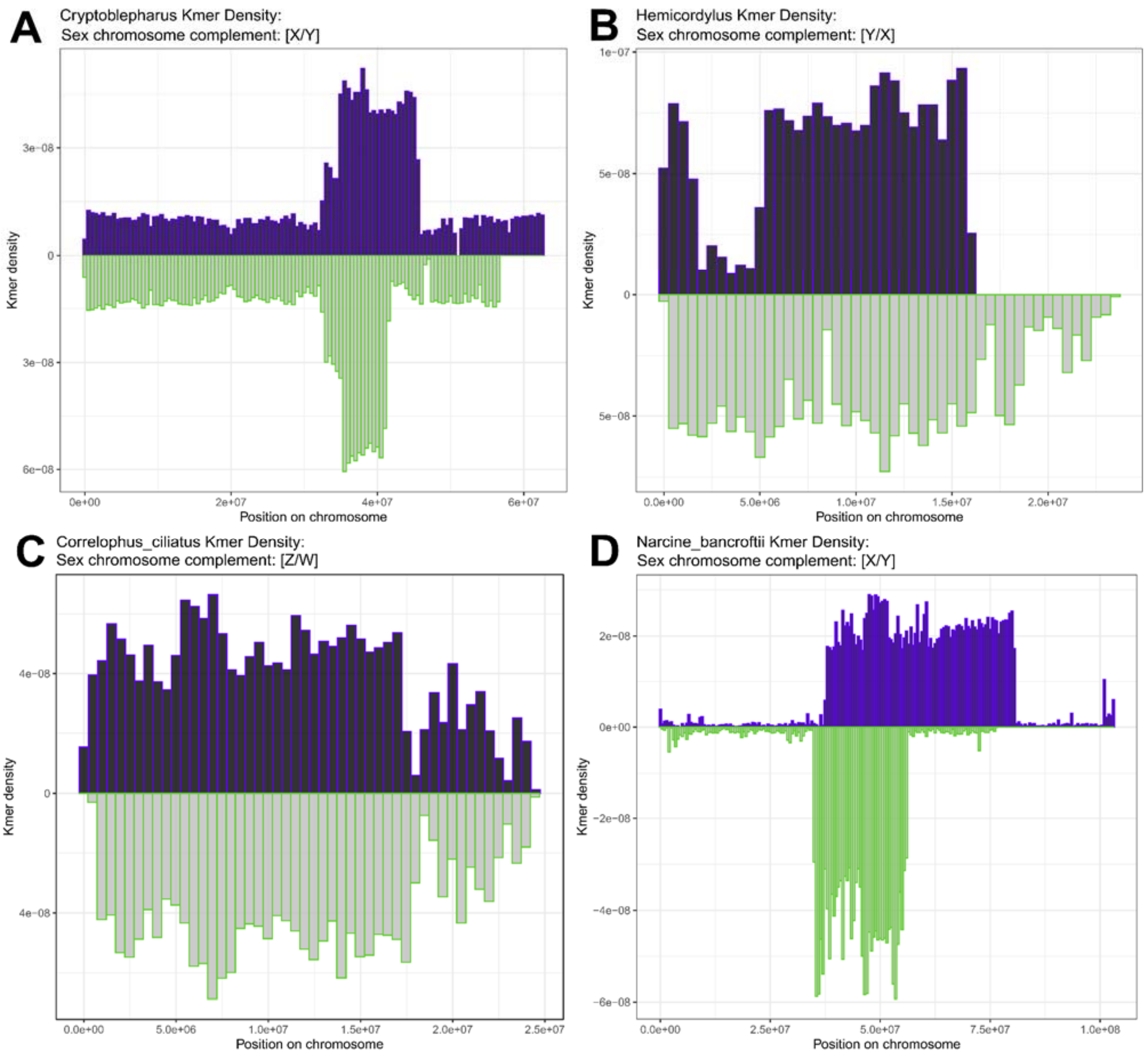

bioRxiv preprint doi: https://doi.org/10.1101/2025.07.07.660342; this version posted July 10, 2025. The copyright holder for this preprint (which was not certified by peer review) is the author/funder, who has granted bioRxiv a license to display the preprint in perpetuity. It is made available under aCC-BY 4.0 International license.

Supplemental Figure 5: Single sex-only mirror plots (part 2).

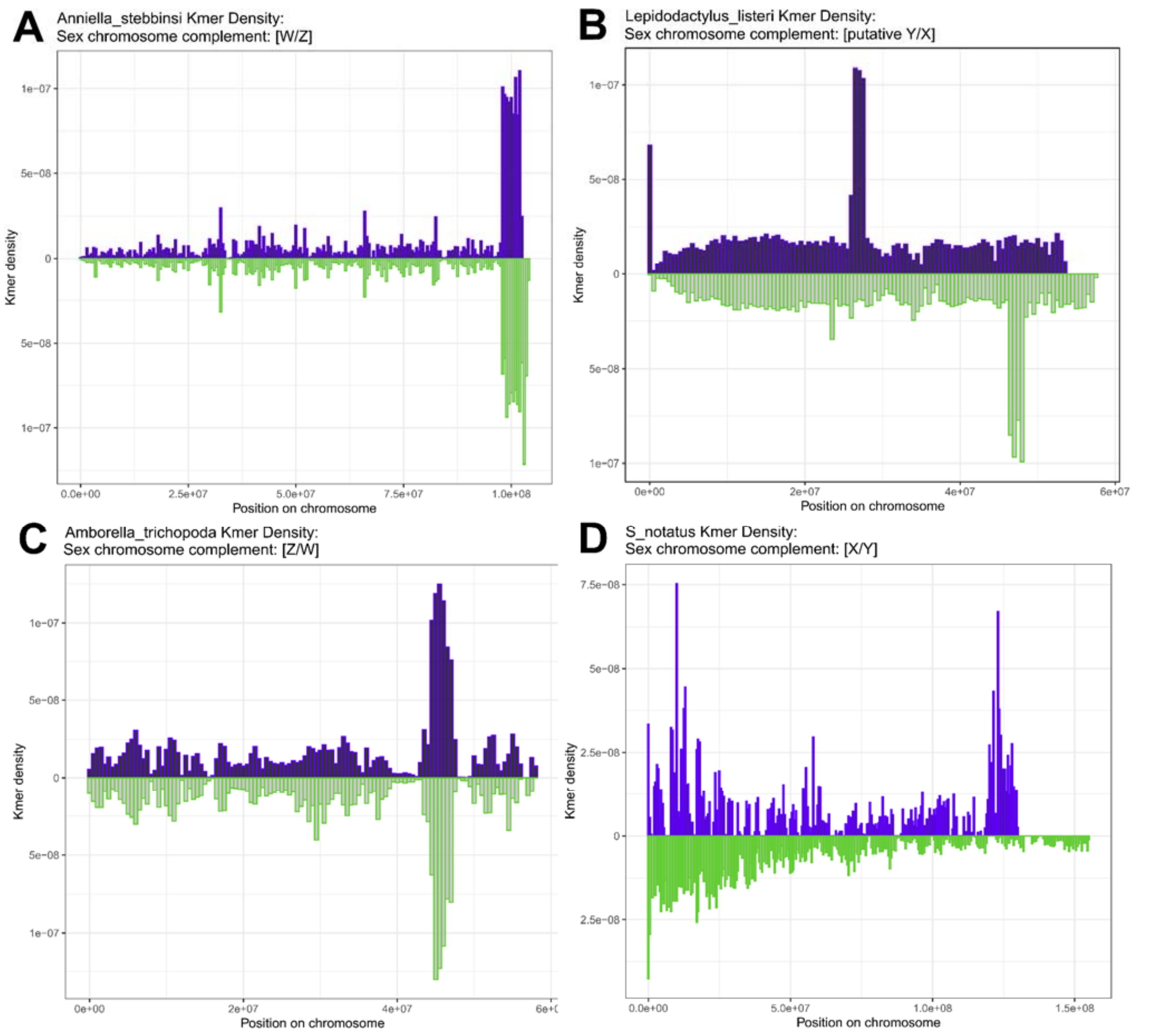

Supplement: Supplement 1 [file NIHPP2025.07.07.660342v1-supplement-1.pdf]
